# Supplementary material for: Perceptions of Antenatal Exercise in Pregnant Females and the Impact of COVID-19
Source: Int J Environ Res Public Health. 2022 Aug 26;19(17):10635. doi: 10.3390/ijerph191710635 (PMC9518533; doi:10.3390/ijerph191710635)
Supplement: Supplementary file 1 [file ijerph-19-10635-s001.zip › ijerph-1819034-supplementary.pdf]

LIVERPOOL JOHN MOORES UNIVERSITY SEMI-STRUCTURED INTERVIEW

1. **Can you tell me about your previous experiences of exercise prior to pregnancy?**

    a. Prompting about any main barriers and benefits etc.

    b. What impact, if any, do you think your regular occupation has on your overall level of physical activity

    c. What did you find difficult about exercise before pregnancy? Prompt to explore further

2. **I'd like to move on to talk a bit about your experiences of pregnancy throughout lockdown?**

    a. At what week of pregnancy did you begin self-isolating?

        i. Prompts about their experience of this , what they found difficult.

    b. Tell me about the way that lockdown impacted your daily life.

    c. What was the main or most difficult change?

        i. Prompts about any difficulties with social-connection/isolation.

        ii. Prompts about changes in occupation, whether they are able to work from home.

    d. Thinking about your experiences of lockdown, how did this impact upon your exercise routine and diet?

    e. What has your experience of pregnancy throughout lockdown been like overall?

    f. Tell me about your experience of healthcare during pregnancy?

        i. Different to/ same as before?

        ii. What impact, if any has this had on your experience of being pregnant?

        iii. What would you have liked instead/ in addition?

3. **Can you tell me about your perceptions of exercise during pregnancy?**

    a. In what way do you feel that advice from your midwife/health care professionals influence your decision to perform exercise during pregnancy?

    b. Motivation – what is motivating you/could help to motivate you?

    c. Support from family/friends?

    d. Challenges – what challenges do you forecast during your pregnancy?

    e. How will you overcome these?

    f. Thinking about your previous experiences of exercise, in what way – if at all – do you feel these impact upon your decisions to continue/stop exercise in pregnancy? Have your previous activity levels influenced your decision to continue/stop exercise in pregnancy?

4. **Can you tell me about any goals you might have in mind that you would like to achieve during your pregnancy?**

    a. Health related/exercise related?

    b. Future intensions post pregnancy?

5. **Can you tell me about how you are feeling about preparing for labour?**

    a. Prompts about birth plan, any specific preparation (e.g. NCT).

6. **Is there anything else relating to this [interview] that you would like to add?**
